# Surgical Management of Extensive Urine Extravasation Resulting in Urethrocutaneous Fistulation and Soft Tissue Necrosis of the Femoral Region Secondary to Pelvic Urethral Rupture in Two Cats

**DOI:** 10.3390/ani16172782

**Published:** 2026-09-04

**Authors:** Dongwoo Kim, Gyeong-Jin Koo, Sang-Kwon Lee, Yoonhoi Koo, Min Jang, Sung-Ho Yun, Young-Sam Kwon, Jinsu Kang

**Affiliations:** 1 Department of Veterinary Surgery, College of Veterinary Medicine, Kyungpook National University, 80, Daehak-ro, Daegu 41566, Republic of Korea; eastox6490@gmail.com (D.K.); sofdiseo@naver.com (G.-J.K.); jangmin@knu.ac.kr (M.J.); shyun@knu.ac.kr (S.-H.Y.); kwon@knu.ac.kr (Y.-S.K.); 2 Department of Veterinary Medical Imaging, College of Veterinary Medicine, Kyungpook National University, 80, Daehak-ro, Daegu 41566, Republic of Korea; sklee10@knu.ac.kr; 3 Department of Veterinary Emergency and Critical Care, College of Veterinary Medicine, Kyungpook National University, 80, Daehak-ro, Daegu 41566, Republic of Korea; yoonhoi@knu.ac.kr

**Keywords:** urine extravasation, urethrocutaneous fistulation, soft tissue necrosis, pelvic urethral rupture, urethrostomy, cat

## Abstract

Pelvic urethral rupture is an uncommon but serious injury in cats that may result in extensive urine extravasation and severe soft tissue damage. This report describes the surgical management of two cats with pelvic urethral rupture complicated by urine extravasation extending into the femoral region, resulting in urethrocutaneous fistulation and extensive soft tissue necrosis. Two 2-year-old male domestic shorthairs presented with similar injuries, one undergoing retrograde urethrography, the other additional CT scan as well to define the extent of injury. In Case 1, transpelvic urethrostomy was performed following surgical debridement of necrotic tissue, and tension-relieving mesh incisions were subsequently made in the surrounding skin of the left thigh to reduce tension on the primary closure and facilitate closure of the skin defect created by debridement. In Case 2, prepubic urethrostomy and surgical debridement were performed following confirmation of pelvic urethral rupture, with contrast accumulation identified at the dorsal aspect of the ischiatic arch on CT. In both cases, the soft tissue wounds were managed by second-intention healing. Both cats recovered without major postoperative complications, and the soft tissue injuries had resolved successfully during follow-up. Both cases demonstrate that prompt surgical intervention combined with appropriate wound management can result in favorable outcomes in cats with pelvic urethral rupture complicated by extensive urine extravasation into the femoral region.

## Simple Summary

Pelvic urethral rupture is a serious injury in cats that can cause urine to leak into the surrounding tissues. When urine spreads into the hindlimb region, it can damage the skin and underlying soft tissues, leading to tissue death and the formation of abnormal openings through which urine drains. This report describes two young cats with this uncommon complication. Both cats underwent surgery to remove damaged tissue and reconstruct the urinary outflow using different surgical techniques according to the location and severity of the injury. The wounds were left to heal on their own with medical management without any further surgery. Both cats recovered without major complications, and the damaged soft tissues healed successfully. These cases demonstrate that severe tissue injury caused by urine leakage into the femoral region can be successfully managed with prompt surgical treatment and careful wound management. Early recognition and appropriate surgical intervention are important for achieving a good clinical outcome in cats with pelvic urethral rupture.

## 1. Introduction

Urine extravasation following urethral injury is uncommon in cats but may result in severe soft tissue damage requiring aggressive medical and surgical management [1]. Urethral injury is most commonly associated with iatrogenic urethral catheterization in male cats with obstructive feline lower urinary tract disease (FLUTD), although pelvic fractures, urethral obstruction secondary to urolithiasis, and external trauma have also been reported [1,2,3,4,5]. In cases of catheter-associated injury, the distal penile urethra and the ischiatic arch are the most frequently affected sites, whereas traumatic injury may result in more extensive urethral disruption [6]. Penile or postprostatic urethral rupture may result in urine leakage and swelling of the perineal region and tail base, while more proximal urethral injury may lead to uroabdomen and peritonitis [1,6].

Diagnosis is based on clinical findings and imaging studies, with perineal swelling, urine leakage, and urinary dysfunction being common clinical findings. Plain radiography may reveal characteristic perineal soft tissue changes associated with urethral rupture, while retrograde positive-contrast urethrography can be used to confirm the presence, location, and extent of urethral disruption [5,6]. In cases with extensive contrast extravasation or severe soft tissue injury, surgical exploration may also be necessary to further assess the extent of urethral damage [6].

Treatment depends on the location and severity of the urethral injury and may include urinary diversion, primary urethral repair, catheter-assisted primary alignment, or urethrostomy [2,6]. Primary alignment using an indwelling urethral catheter has been described for traumatic urethral rupture in cats [2], whereas more extensive defects may require resection and anastomosis or salvage urethrostomy [6]. When extensive urine-induced tissue necrosis precludes primary wound closure, reconstructive techniques such as local rotational or axial pattern skin flaps may be required [1]. Vacuum-assisted closure (VAC) therapy has also been reported as an effective option for managing severe urine-induced soft tissue necrosis before definitive wound reconstruction [7]. Postoperative evaluation should include monitoring of urinary function and wound healing, as well as surveillance for complications such as urethral stricture, recurrent urinary tract infection, urinary incontinence, and urethrocutaneous fistulation [3,4,6]. Previous studies have evaluated clinical outcomes through postoperative follow-up and assessment of urinary function and treatment-related complications over several months [3,4].

This report describes two cases of pelvic urethral rupture in which urine extravasation extended into the hindlimb soft tissues, resulting in severe thigh skin necrosis and urethrocutaneous fistulation. The aim of this report is to highlight an unusual pattern of urine distribution following urethral injury and to describe the surgical management of these injuries using different urethrostomy techniques combined with soft tissue reconstruction and wound management.

## 2. Case Presentation

### 2.1. Case 1

A 2-year-old male castrated domestic shorthair cat was presented with acute dysuria, hematuria, and azotemia secondary to suspected urethral obstruction. No uroliths or other intraluminal obstructive material were identified, and the underlying cause of the urethral obstruction could not be definitively determined at the time of initial presentation. The clinical findings were considered compatible with lower urinary tract disease. Temporary urethral catheterization was performed under sedation with butorphanol (Butophan; Myungmoon Pharm., Seoul, Republic of Korea) (0.3 mg/kg, IV) using a Tomcat urinary catheter (BUSTER Cat Catheter, KRUUSE, Langeskov, Denmark) to restore urinary outflow. The catheter was maintained for 2 days, then removed, and the patient was discharged following stabilization. Recurrent urethral obstruction occurred 10 days later and was managed in a similar fashion.

**Figure 1:**
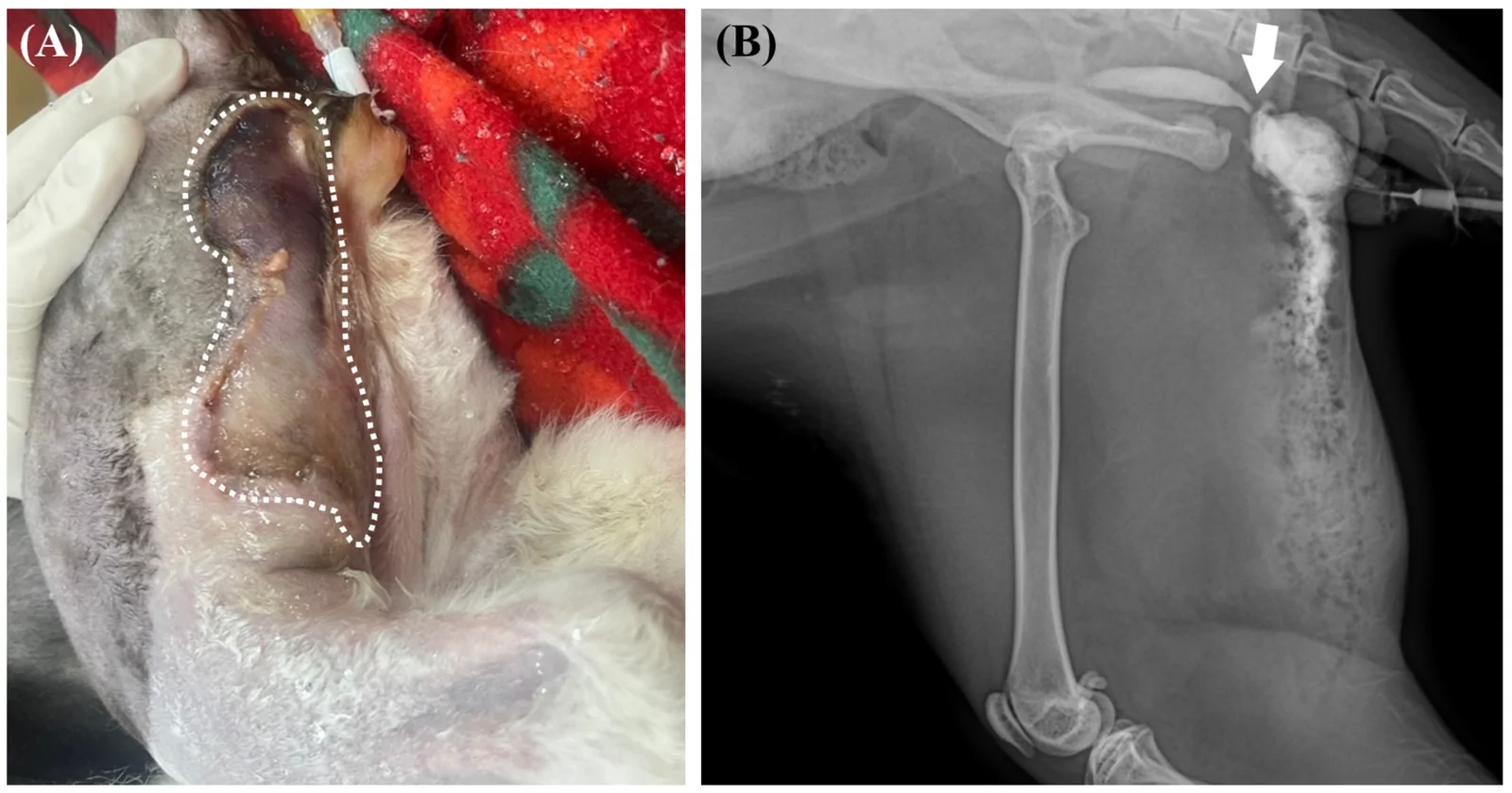
Clinical presentation and diagnostic imaging findings in Case 1. (**A**) Progressive necrosis of the left thigh associated with urine extravasation (area outlined by the dashed line) following suspected urethral injury. Extensive cutaneous and subcutaneous tissue damage is evident over the lateral aspect of the thigh. (**B**) Positive-contrast retrograde urethrography demonstrating extravasation of contrast medium (white arrow) into the pelvic soft tissues caudal to the ischiatic table, confirming postprostatic urethral rupture.

Approximately 1 week after the second intervention, progressive cutaneous necrosis was noted over the caudodorsal aspect of the left thigh, extending from the femoral region toward the area near the stifle joint, raising suspicion of urethral rupture with urine extravasation (Figure 1A). The cat remained clinically stable and showed no apparent lameness. Straining during urination was not observed, as urine was able to drain through a urethrocutaneous fistula and subsequently accumulate within the subcutaneous tissues of the affected thigh. Retrograde urethrography demonstrated contrast medium (iohexol) (Omnipaque, GE HealthCare, Chicago, IL, USA) leakage into pelvic soft tissues caudal to the ischiatic table, consistent with post-prostatic urethral rupture (Figure 1B). Surgical exploration was planned to debride necrotic tissue and identify and repair the ruptured urethra.

**Figure 2:**
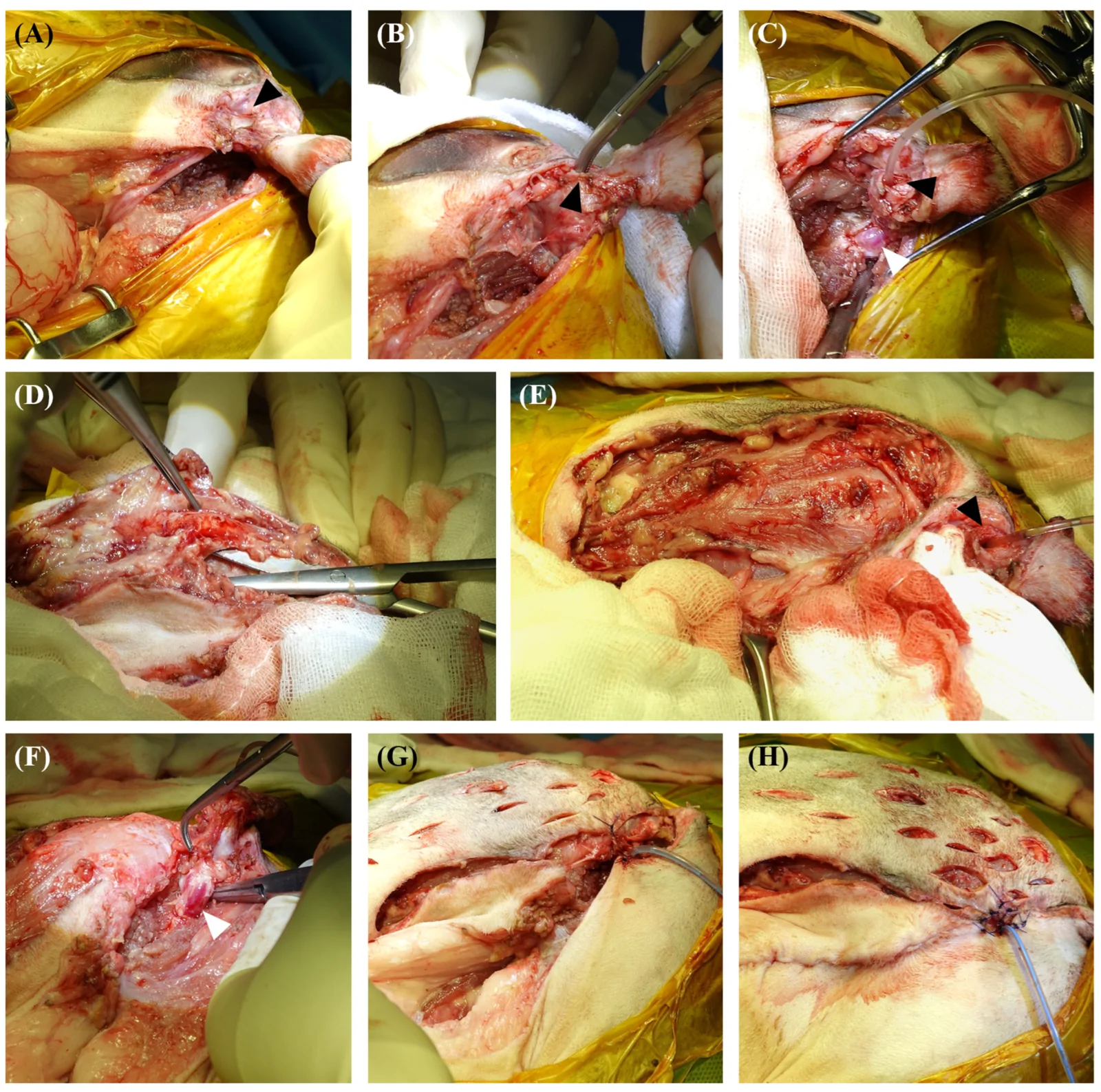
Surgical management of Case 1. (**A**,**B**) Intraoperative identification of extensive pelvic urethral rupture with urethrocutaneous fistulation extending into the left hindlimb. (**C**) Exposure of the postprostatic urethra following partial pubic ostectomy. (**D**,**E**) Debridement of necrotic soft tissues extending from the urethral rupture site to the femoral region. (**F**) Identification and mobilization of a healthy urethral segment following debridement. (**G**,**H**) Creation and completion of a tension-free urethrostomy with a 6-Fr Foley balloon urinary catheter maintained within the urethral lumen during apposition of the urethral mucosa to the surrounding skin using simple interrupted sutures. Black arrowheads indicate the cutaneous opening of the urethrocutaneous fistula, whereas white arrowheads indicate the postprostatic urethra.

A ventral midline celiotomy was performed, and the pelvic urethra was approached via a caudal abdominal incision. Extensive urethral rupture with urethrocutaneous fistulation extending into the left hindlimb was identified (Figure 2A,B). Following partial pubic ostectomy to expose the postprostatic urethra (Figure 2C), necrotic tissue extending from the urethral rupture site to the femoral region was debrided using a combination of sharp and blunt dissection until viable tissue was identified (Figure 2D,E). Following debridement, a healthy segment of the post-prostatic urethra proximal to the rupture site was identified. The ischiocavernosus and ischiourethralis muscles were released from their insertions on the ischium to facilitate urethral mobilization, and the urethra was subsequently transected at a level that allowed creation of a tension-free urethrostomy. (Figure 2F). The urethra was transected at a level that allowed creation of a tension-free urethrostomy. A 6-Fr Foley balloon urinary catheter (All Silicone Foley Balloon Catheter, Sewoon Medical Co., Ltd., Cheonan, Republic of Korea) was placed within the urethral lumen, and the ventral urethral wall was incised to create a urethrotomy. The catheter was maintained for 4 days postoperatively to provide temporary urinary diversion during the early postoperative period and was subsequently removed. After debridement of the necrotic tissue on the medial aspect of the left thigh, the surrounding healthy skin was mobilized and advanced to the urethral mucosa for primary apposition. The skin was sutured to the urethral mucosa using 4-0 polydioxanone (PDS; Ethicon, Cincinnati, OH, USA) in a simple interrupted pattern to create a new stoma. Because excessive tension was present on the surrounding skin following primary apposition, tension-relieving mesh incisions were subsequently made in the surrounding skin of the left thigh. Individual skin incisions were approximately 0.5–1 cm in length and were placed according to the local tissue defect to reduce excessive tension on the closure and facilitate wound closure (Figure 2G,H).

**Figure 3:**
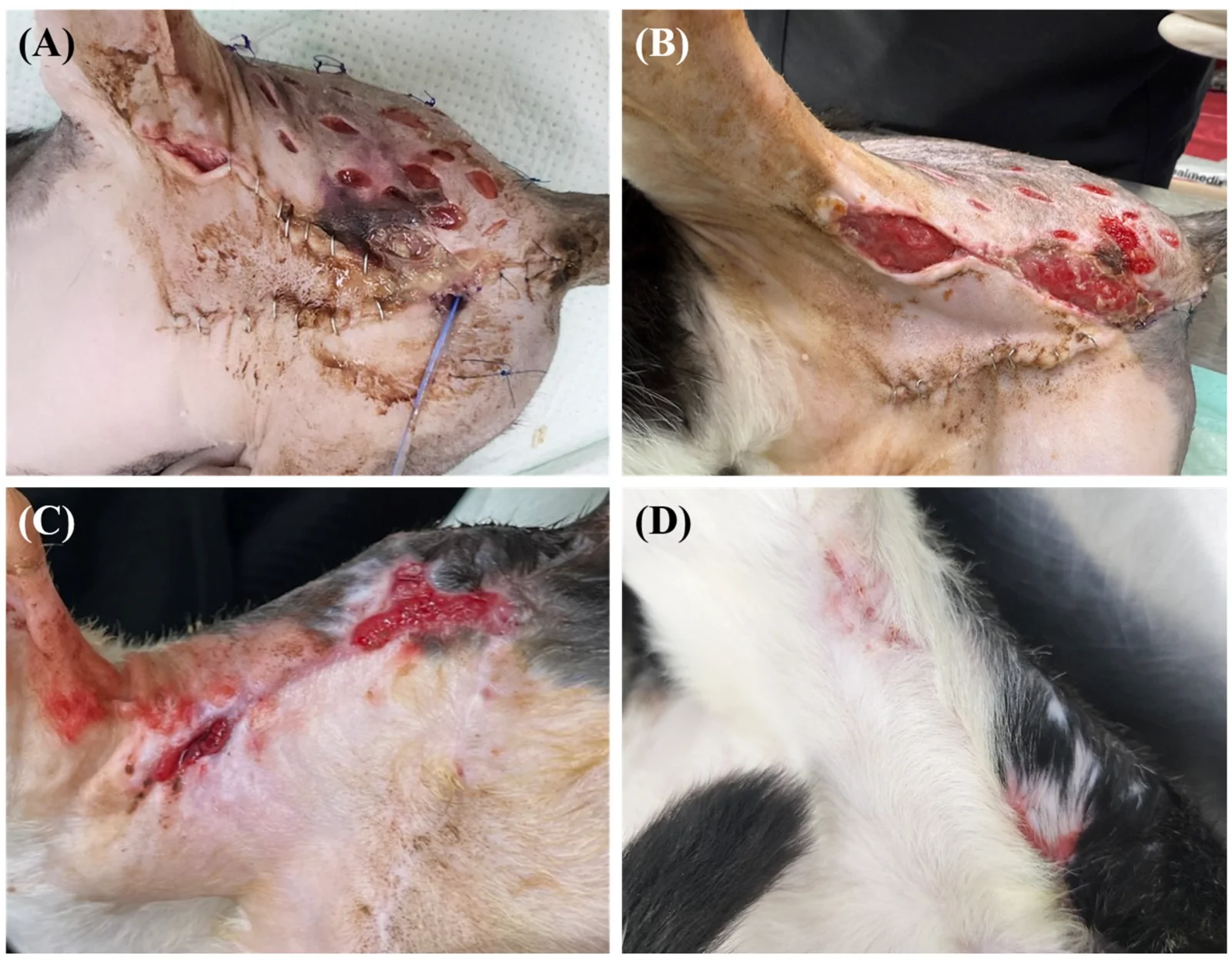
Postoperative wound healing in Case 1. (**A**) A total of 4 days postoperatively. (**B**) A total of 8 days postoperatively. (**C**) A total of 27 days postoperatively. (**D**) A total of 39 days postoperatively, demonstrating complete healing with normal hair regrowth and urination.

Postoperatively, localized skin necrosis developed in a portion of the wound associated with the tension-relieving mesh incisions. The necrotic area was located away from the stoma and did not substantially affect the urethrostomy. The necrotic tissue was debrided, and the wound was managed conservatively with a tie-over bandage and 100% medical-grade Manuka honey (Activon Tube; Brightwake Ltd., Nottingham, UK) for the first 5 postoperative days. As healthy granulation tissue developed, honey therapy was discontinued, and the wound was subsequently managed with a self-adherent soft silicone foam dressing (Mepilex Border Post-Op; Mölnlycke Health Care, Gothenburg, Sweden). The cat was hospitalized for 14 days after surgery. The 6-Fr Foley balloon catheter was removed on postoperative day 4, after which the cat urinated spontaneously without difficulty. No clinical signs of cystitis were observed during hospitalization or subsequent follow-up. At 39 days postoperatively, the cat was re-evaluated as part of a routine follow-up examination, which confirmed complete wound healing with normal hair regrowth and normal urination (Figure 3). During 20 months of follow-up, two episodes of mild stomal narrowing occurred at 3 and 6 months postoperatively, respectively. Both episodes were transient and successfully managed with temporary catheterization maintained for 2 days on each occasion, without the need for revision surgery. No clinical signs suggestive of FLUTD or cystitis were observed during these episodes. Based on the transient nature of the narrowing and its resolution following temporary catheterization, the episodes were considered more consistent with temporary stomal narrowing than recurrent FLUTD or cystitis. The surgical site remained otherwise unremarkable.

### 2.2. Case 2

A 2-year-old male domestic shorthair cat was presented with a history of chronic urine leakage from a cutaneous defect over the left thigh following an unknown traumatic injury approximately 1 year previously. The cat had been rescued by the owner after the injury, and the specific cause and mechanism of the trauma were unknown. The owner reported that the cutaneous defect intermittently developed crusting and subsequently reopened, with persistent urine leakage through the defect. Although the owner was aware of the urine leakage, definitive treatment could not be pursued for an extended period. The patient remained clinically stable without apparent urinary signs until the recent onset of stranguria, followed by anuria that persisted for more than 1 week.

**Figure 4:**
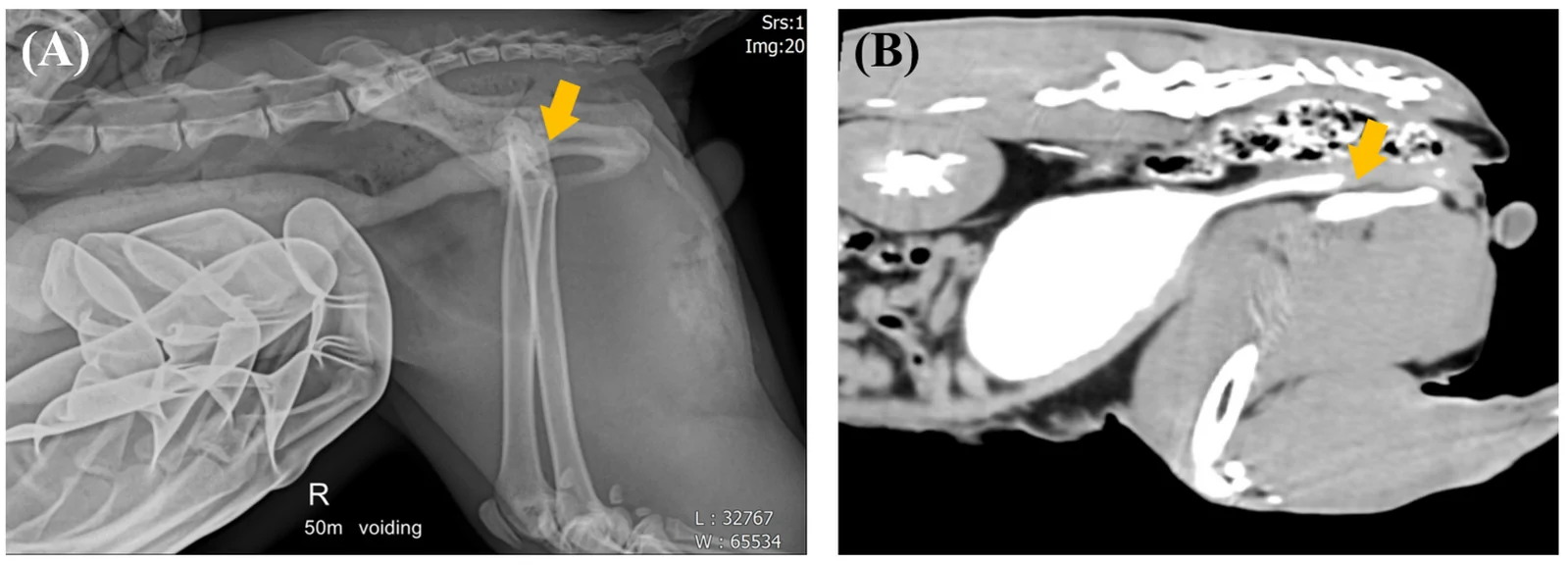
Diagnostic imaging of the pelvic urethral rupture in Case 2. (**A**) Voiding urethrography performed after intravenous administration of contrast medium and subsequent filling of the urinary bladder with contrast medium, followed by bladder compression, demonstrating diffuse contrast extravasation caudal to the ischium (orange arrowhead). (**B**) Computed tomography image showing contrast accumulation dorsal to the ischiatic arch (orange arrow).

Voiding urethrography was performed after intravenous administration of contrast medium (iohexol) (Omnipaque, GE HealthCare, Chicago, IL, USA) and subsequent filling of the urinary bladder with contrast medium. The urinary bladder was manually compressed through gentle external abdominal pressure to induce voiding, during which diffuse contrast extravasation was observed caudal to the ischium, consistent with pelvic urethral rupture (Figure 4A). Because the pelvic bones limited precise localization of the urethral rupture on radiographic imaging, computed tomography (CT) (Alexion16 TSX-034A, Toshiba, Kawasaki, Japan) demonstrated contrast accumulation at the dorsal aspect of the ischiatic arch, further supporting the diagnosis of pelvic urethral rupture (Figure 4B). Surgery was planned to debride necrotic tissue and repair the urethral rupture.

**Figure 5:**
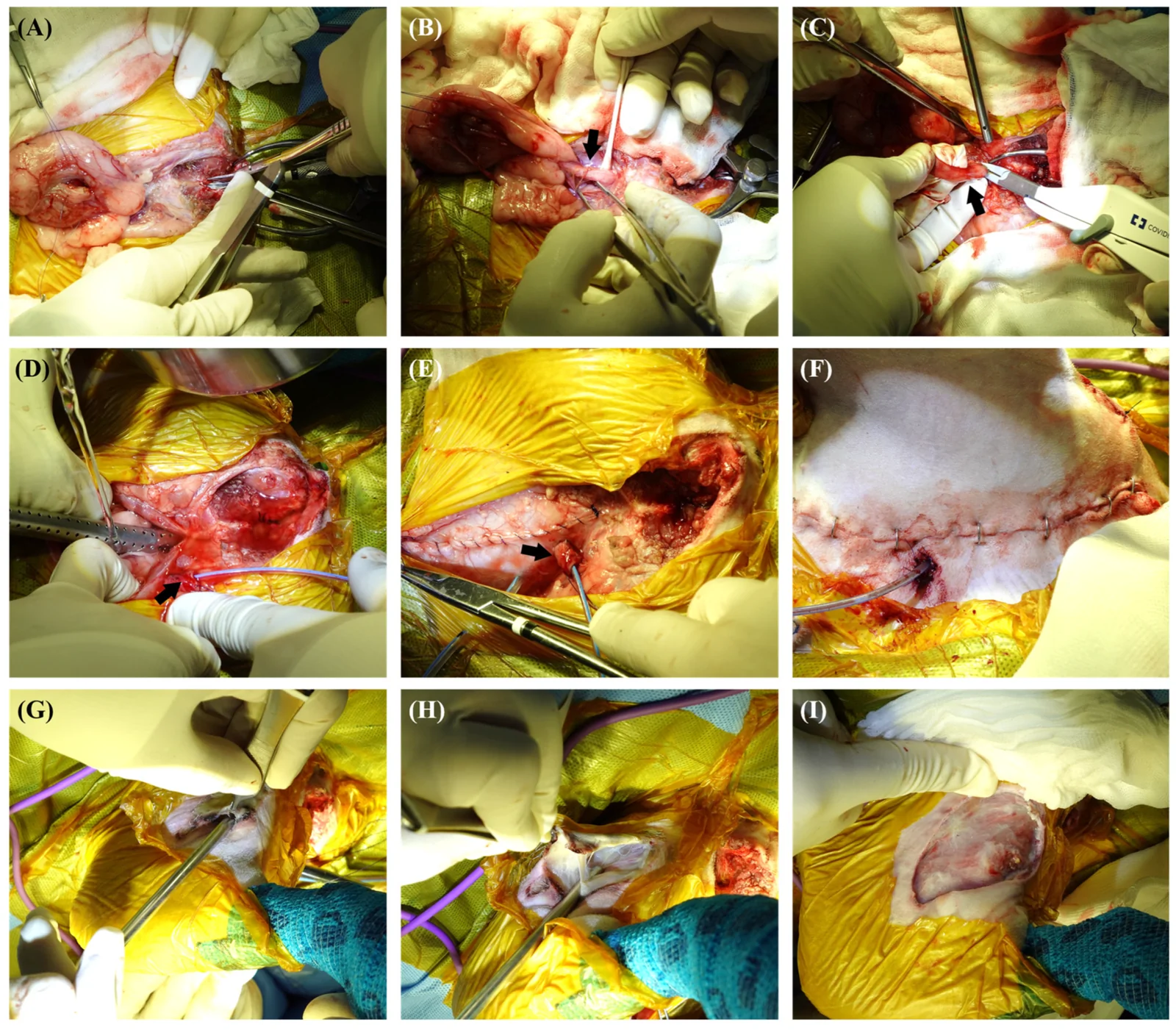
Surgical management of Case 2. (**A**) Ventral midline celiotomy and cystotomy for placement of a 5-Fr feeding tube through the urinary bladder and urethra to assess the location and extent of the urethral stricture, followed by exposure of the pelvic urethra after partial pubic ostectomy. (**B**,**C**) Mobilization of the pelvic urethra and transection at a level of healthy urethra proximal to the rupture site. (**D**,**E**) Exteriorization of the urethra through the caudal abdominal incision, followed by placement of a new balloon urinary catheter through the urethral lumen and advancement into the urinary bladder. (**F**) Completion of the prepubic urethrostomy. (**G**–**I**) Surgical debridement was performed for extensive skin necrosis and soft tissue injury involving the left thigh and femoral region secondary to urine extravasation. Black arrows indicate the pelvic urethra.

A ventral midline celiotomy was performed, and a cystotomy was made to allow placement of a 5-Fr feeding tube into the urinary bladder. The feeding tube was advanced through the bladder and urethra to assess the location and extent of the urethral stricture (Figure 5A). After the location and extent of the urethral stricture were confirmed, the feeding tube was removed, and the cystotomy site was closed with 3-0 polydioxanone (PDS; Ethicon, Cincinnati, OH, USA) using a simple interrupted suture pattern before proceeding with urethral reconstruction. Following exposure of the pelvic urethra via partial pubic ostectomy, a transpelvic urethrostomy (TPU) was attempted; however, it could not be completed because of insufficient urethral length and excessive caudal tension (Figure 5B). The procedure was therefore converted to a prepubic urethrostomy (PPU). The pelvic urethra was mobilized and transected distal to healthy tissue (Figure 5C). The urethra was subsequently exteriorized through the caudal abdominal incision, and a new balloon urinary catheter was inserted through the urethral lumen and advanced into the urinary bladder (Figure 5D–F). Necrotic tissue associated with urine extravasation involving the left thigh and extending to the femoral region was debrided, and the wound was closed routinely (Figure 5G–I).

**Figure 6:**
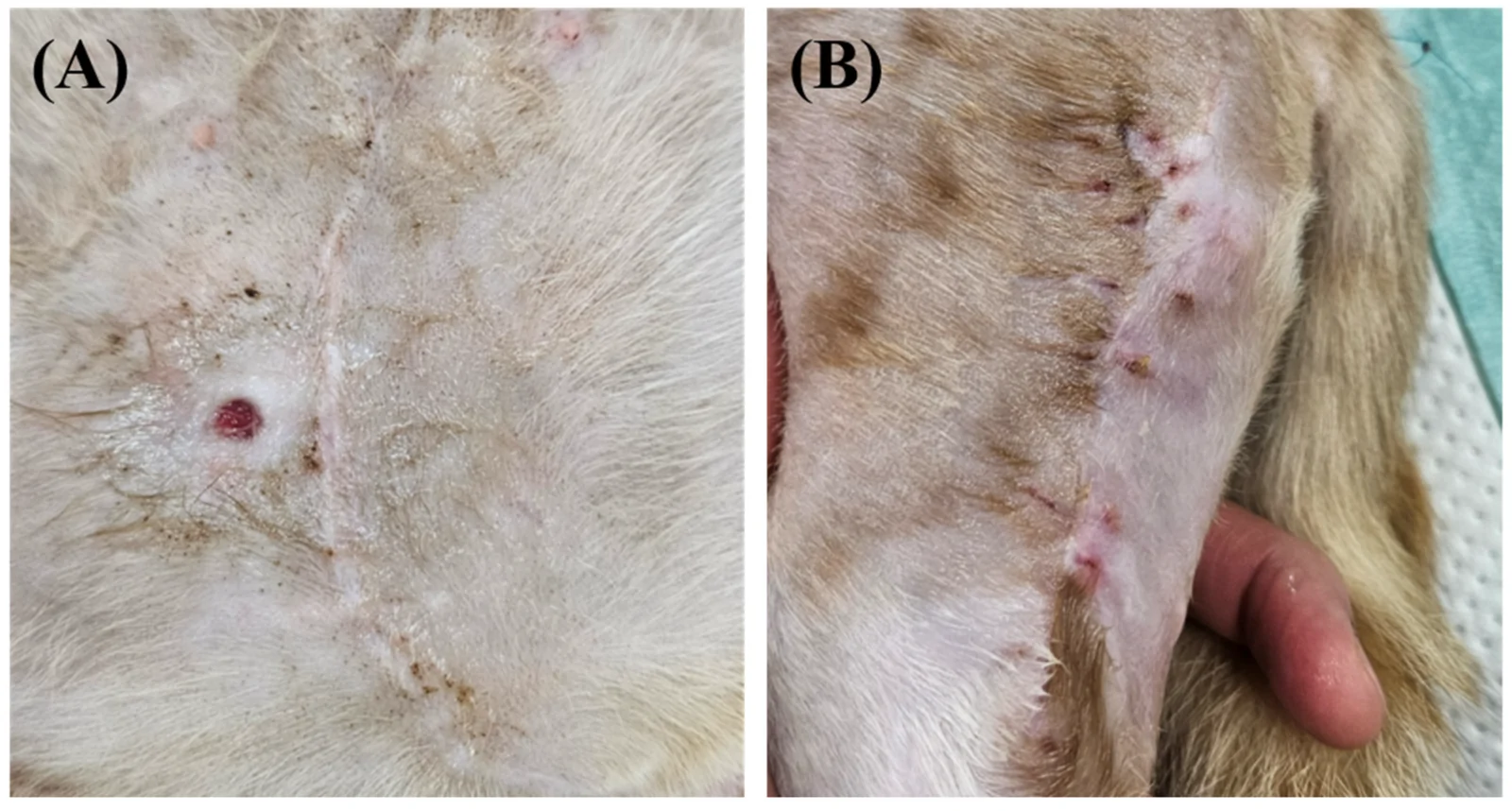
One-month postoperative outcome in Case 2. (**A**) Appearance of the prepubic urethrostomy stoma. (**B**) Healed wound in the femoral region.

Postoperatively, azotemia resolved with improved renal parameters. Amoxicillin–clavulanate (Amocla Tablet 375 mg; Kuhnil Pharm., Seoul, Republic of Korea) (12.5 mg/kg, PO, q12h) was administered for 14 days for antimicrobial therapy, and meloxicam (METACAM^®^; Boehringer Ingelheim Animal Health USA Inc., Duluth, GA, USA) (0.1 mg/kg, PO, q24h) was administered for 3 days for postoperative analgesia. Partial wound dehiscence occurred approximately 1 week after surgery but healed by second intention without intervention. At 1-month follow-up, the surgical site was unremarkable (Figure 6). The patient was re-evaluated at 3 months postoperatively to assess urinary function and the surgical site. No apparent urinary incontinence was observed during hospitalization or follow-up, and the owner did not report any episodes of urinary incontinence. Subsequent follow-up was conducted by telephone. The cat remained clinically stable at 15 months.

## 3. Discussion

While previously reported patterns of urine extravasation are generally confined to the perineal region and tail base, and more proximal rupture may result in uroabdomen [6], extension into the hindlimb soft tissues has rarely been documented in veterinary literature [8,9]. In the two cases reported here, urine extravasation extended beyond the pelvic and perineal regions into the hindlimb soft tissues, reaching the distal femoral region and resulting in marked thigh skin necrosis and urethrocutaneous fistulation, an uncommon manifestation of pelvic urethral rupture.

Although rarely reported, urine may track along continuous fascial planes between the pelvic canal and hindlimb musculature following pelvic urethral disruption [10], which is considered the most likely mechanism in the present cases. Urine extravasation within tissue planes may result in inflammation, tissue necrosis and delayed wound healing [5]. In Case 2, a chronic cutaneous defect following previous trauma may also have facilitated formation of a persistent urethrocutaneous fistula, contributing to continued urine leakage and delayed presentation.

Both cases highlight the diagnostic challenge associated with pelvic urethral rupture when clinical signs are dominated by extra-urinary manifestations. Progressive thigh skin necrosis may be misinterpreted as a primary dermatological or traumatic condition, potentially delaying diagnosis [5,11]. Importantly, the ability to urinate or pass a urinary catheter does not exclude significant urethral injury [6,11]. In these cases, urinary leakage through a urethrocutaneous fistula may have provided an alternative pathway for urine drainage, potentially masking the severity of the underlying urethral injury. This may explain why marked urinary obstruction was not evident despite extensive urethral disruption and soft tissue necrosis. Positive-contrast urethrography therefore remains essential for confirming urethral disruption and localizing urine extravasation [11]. In Case 2, CT further aided surgical planning by defining the anatomical relationship between the urethral rupture and the ischiatic arch, which was difficult to delineate on radiography because of superimposition of the pelvic bones.

Surgical management should be tailored to the location and extent of urethral injury and the ability to create a tension-free urinary stoma. TPU was successfully performed in Case 1 following urethral mobilization and partial ischial ostectomy. In Case 2, insufficient urethral length prevented tension-free TPU despite caudal ischiectomy and urethral dissection, necessitating conversion to a PPU. Because excessive tension increases the risk of wound dehiscence, urine leakage, stomal stricture formation and surgical failure, PPU remains an important salvage option when adequate urethral length or mobility cannot be achieved [12,13,14]. In addition, in cases of extensive and severe skin and subcutaneous tissue necrosis around the penis caused by urine leakage, PPU rather than TPU may be considered, regardless of tissue tension, as it allows apposition of healthy skin to healthy urethral tissue and may facilitate healing of the necrotic area. These cases emphasize the importance of intraoperative assessment, as definitive selection of urethrostomy technique may depend on findings that cannot be fully predicted preoperatively.

Previous reports describing urine extravasation-associated soft tissue necrosis in cats have relied on rotational or axial pattern flaps to reconstruct extensive defects involving the perineal region and tail base [1]. In contrast, flap reconstruction was not required in either case. Wound closure was achieved using tension-relieving mesh incisions followed by second-intention healing in Case 1 and primary closure in Case 2. These findings suggest that, when viable tissue can be identified after adequate debridement and sufficient local skin mobility is available, extensive urine-induced thigh wounds may be managed successfully without more complex reconstructive procedures. This may reflect greater skin mobility and tissue availability in the thigh compared with the perineal region [10]. Both cases achieved satisfactory wound healing and favorable long-term outcomes.

Postoperative complications associated with urethrostomy include stomal narrowing, incontinence and recurrent urinary tract infection. Only mild transient stomal narrowing occurred in Case 1 and was managed conservatively. Long-term outcomes in both cats were favorable, with satisfactory urinary function and complete wound healing achieved.

These cases demonstrate that pelvic urethral rupture should be considered in cats presenting with unexplained soft tissue necrosis, particularly in the presence of concurrent urinary tract disease or trauma. They further highlight that urine extravasation may extend beyond classical anatomical compartments, resulting in clinically significant hindlimb involvement. Early diagnosis, appropriate urinary diversion, and aggressive wound management are essential to achieve favorable outcomes.

The main limitation of this report is the small number of cases. In Case 1, antegrade urethrocystography was not performed, and therefore, more proximal urethral injuries may have been overlooked. In addition, the proposed mechanism of urine extravasation extending beyond the pelvic region into the hindlimb soft tissues and femoral region was inferred from anatomical considerations and clinical findings rather than direct confirmation. Consequently, the exact route of dissemination remains speculative. Further reports and advanced imaging investigations may help clarify the pathogenesis of this unusual presentation.

## 4. Conclusions

In conclusion, pelvic urethral rupture should be considered as a differential diagnosis in cats presenting with unexplained thigh skin necrosis or urethrocutaneous fistulation following trauma, even when urination is preserved or urethral catheterization is possible. These cases demonstrate that urine extravasation may extend beyond the classical perineal region into the hindlimb soft tissues, resulting in extensive tissue injury and delayed diagnosis. Successful management depends on early recognition, accurate imaging to define the extent of urethral injury and urine extravasation, appropriate selection of urinary diversion based on intraoperative findings, and aggressive wound management. When sufficient urethral length or tissue mobility cannot be achieved, or when extensive periurethral skin and subcutaneous necrosis is present, PPU may be considered as an alternative to TPU to facilitate tension-free apposition of healthy urethral and cutaneous tissues and promote wound healing. Increased awareness of this uncommon presentation may facilitate earlier diagnosis and improve clinical outcomes in affected cats.

## Data Availability

The original contributions presented in this study are included in the article. Further inquiries can be directed to the corresponding author.

## Abbreviations

The following abbreviations are used in this manuscript:

FLUTD	Feline lower urinary tract disease
TPU	Transpelvic urethrostomy
CT	Computed tomography
PPU	Prepubic urethrostomy
